# Impact of mass drug administration of azithromycin for trachoma elimination on prevalence and azithromycin resistance of genital *Mycoplasma genitalium* infection

**DOI:** 10.1136/sextrans-2018-053938

**Published:** 2019-04-13

**Authors:** Mark Andrew Harrison, Emma Michele Harding-Esch, Michael Marks, Marcus James Pond, Robert Butcher, Anthony W Solomon, Liqing Zhou, NgeeKeong Tan, Achyuta V Nori, Henry Kako, Oliver Sokana, David C W Mabey, Syed Tariq Sadiq

**Affiliations:** 1 Applied Diagnostic Research and Evaluation Unit, St George's, University of London, London, UK; 2 HIV/STI Department, Public Health England, London, UK; 3 Clinical Research Department, London School of Hygiene and Tropical Medicine, London, UK; 4 Southwest London Pathology, St George's University Hospitals NHS Foundation Trust, London, UK; 5 Department of STI and HIV Prevention, Ministry of Health and Medical Services, Honiara, Solomon Islands; 6 Eye Health Department, Ministry of Health and Medical Services, Honiara, Solomon Islands

**Keywords:** mycoplasma, antimicrobial resistance, trachoma, infection, drug resistance, *Mycoplasma genitalium*, mass drug administration, azithromycin

## Abstract

**Background:**

Mass drug administration (MDA) of 20 mg/kg (maximum 1 g in adults) azithromycin for ocular *Chlamydia trachomatis* (CT) infection is a key component of the WHO trachoma elimination strategy. However, this dose may be suboptimal in *Mycoplasma genitalium* infection and may encourage emergence of antimicrobial resistance (AMR) to azithromycin.

**Objectives:**

To determine the effect of MDA for trachoma elimination on *M. genitalium* prevalence, strain type and azithromycin resistance.

**Methods:**

A secondary analysis of CT-negative vulvovaginal swabs from three outpatient antenatal clinics (Honiara, Solomon Islands) from patients recruited either pre-MDA, or 10 months post-MDA in two cross-sectional surveys was carried out. Swabs were tested for *M. genitalium* infection using Fast Track Diagnostics Urethritis Plus nucleic acid amplification assay. *M. genitalium*-positive samples were subsequently tested for azithromycin resistance by sequencing domain V of the 23S rRNA DNA region of *M. genitalium* and underwent phylogenetic analysis by dual locus sequence typing.

**Results:**

*M. genitalium* prevalence was 11.9% (28/236) in women pre-MDA and 10.9% (28/256) 10 months post-MDA (p=0.7467). Self-reported receipt of azithromycin as part of MDA was 49.2% in women recruited post-MDA and 17.9% (5/28) in those who tested *M. genitalium* positive. Of samples sequenced (21/28 pre-MDA, 22/28 post-MDA), all showed a macrolide susceptible genotype. Strain typing showed that sequence types diverged into two lineages, with a suggestion of strain replacement post-MDA.

**Conclusion:**

A single round of azithromycin MDA in an island population with high baseline *M. genitalium* prevalence did not appear to impact on either prevalence or azithromycin resistance, in contrast to reported decreased genital CT prevalence in the same population. This may be due to limitations such as sample size, including CT-negative samples only, and low MDA coverage. Further investigation of the impact of multiple rounds of MDA on *M. genitalium* azithromycin AMR in antibiotic experienced and naïve populations is warranted.

## Background

*Mycoplasma genitalium* (MG) is an STI of increasing global importance, causing serious maternal and child health sequelae.^1 2^ Management is threatened by antimicrobial resistance (AMR) to the first-line treatment azithromycin,^3^ and treatment options are limited.^4^ Trachoma is the leading infectious cause of blindness worldwide,^5^ caused by ocular *Chlamydia trachomatis* (CT) infection. A key component of the WHO trachoma elimination strategy is mass drug administration (MDA) with 20 mg/kg azithromycin, up to a maximum of 1 g in adults. MDA is recommended annually for a minimum of 3 years in districts where trachoma is endemic (trachomatous inflammation-follicular prevalence ≥10% in 1–9 year-olds).^6^ Temporary increases in carriage of macrolide-resistant *Streptococcus pneumoniae*, *Staphylococcus aureus* and *Escherichia coli* have been observed following MDA.^7^ Thus, while MDA with azithromycin may have significant benefits through reducing prevalence of active trachoma, ocular CT infection,^8^ genital CT infection^9^ and even child mortality,^10^ these may be undermined by subsequent emergence of AMR in target and non-target organisms.

Azithromycin resistance rates vary worldwide, with reports of 79.4% in Australia^11 12^ and over 70% in Japan.^13^ Limited data exist on resistance prevalence in Pacific Basin nations. An estimated 12% of infected patients treated with 1 g azithromycin develop macrolide resistance after treatment^14^ with resistance mediated by single nucleotide polymorphisms (SNP) most frequently observed at adenosine nucleotides at positions 2058 and 2059 within the 23S rRNA gene.^15^ Resistance selecting pressures, such as mass treatment programmes, may worsen this situation. This raises the question of whether secondary beneficial impacts of azithromycin MDA (reduced morbidity and mortality) should be weighed against potential negative impacts (AMR). Given the large populations treated with azithromycin by trachoma elimination programmes (over 800 million doses provided to programmes by the International Trachoma Initiative as of December 2018^16^), we aimed to ascertain the effect of a trachoma programme MDA distribution on MG prevalence and azithromycin resistance.

## Methods

### Study population

Patient samples and data were collected as described previously.^9^ Briefly, women aged 16–49 years attending three community antenatal clinics (ANC) in Honiara, Solomon Islands, were recruited over 10 days in August 2014. Participants were invited to take part pre-MDA. A new group of women aged 16–49 years was enrolled in the same clinics 10 months post-MDA, over 5 days in July 2015. Demographic and clinical data were collected by clinic nursing staff. At both time points, two self-taken vulvovaginal swabs were collected and stored at −20°C in the recruiting clinic; one was tested for CT and *Neisseria gonorrhoeae* using the ProbeTec CT/GC assay (Becton Dickinson, USA) at the Solomon Islands national reference laboratory, while the other was shipped on dry ice to the London School of Hygiene and Tropical Medicine (UK) and stored at −20°C. Swabs matched to CT-negative samples from reference testing were included in this study (CT-positive samples were allocated for other research^9^), and were transported dry on dry ice to St George’s, University of London, UK.

### Pathogen detection

DNA from swabs was eluted in 1 mL phosphate buffered saline (PBS) (Sigma-Aldrich, USA) followed by vortexing for 15 s and brief centrifugation. The entire eluate was removed in preparation for testing. During preparation, one media control sample of PBS was included for every 32 samples. DNA extraction was carried out by the QIASymphony SP/AS instrument (Qiagen, Germany) with the Virus/Pathogen Mini Kit using the Complex 200 protocol, with a 60 µL elution volume. MG was tested for using FTD Urethritis Plus (FTDUP) PCR Kit (Fast-Track Diagnostics, Luxembourg) on the Rotor-Gene Q (Qiagen) according to manufacturer’s instructions. FTDUP positivity was defined as an exponential amplification signal crossing a threshold of 0.05 normalised fluorescence, as per standard practice at South West London Pathology, St George’s University Hospitals NHS Foundation Trust. For a result to be considered valid, (1) the internal control had to be positive and have a cycle threshold (Cq) value ≤33, or if above 33, within ±3.3 Cq of the extraction control’s Cq; and (2) positive, media and no-template controls had to pass. The person carrying out the testing was blind to all patient data, including reference CT/NG test results.

### MG 23S rRNA genotyping

Samples identified as MG positive by FTDUP underwent Sanger sequencing of the domain V region of the 23S rRNA gene to identify SNPs at positions 2058 and 2059 (*E. coli* numbering) associated with high-level macrolide resistance. Two microlitres of extracted DNA was amplified using previously validated primers^15^ using the Multiplex PCR Kit (Qiagen) according to manufacturer’s instructions on a GS-1 thermal cycler (G-Storm, UK). Pre-MDA samples were amplified as follows: 95°C 15 min, 45 cycles of 94°C 30 s, 56.5°C 90 s and 72°C 90 s, with a final step of 72°C 10 min. Post-MDA samples were amplified as follows: 94°C 15 min, 45 cycles of 94°C 30 s, 60°C 3 min and 72°C 90 s with a final step of 72°C 10 min. If the initial 2 µL of extracted DNA used as template failed to generate product, then PCR was repeated with 5 µL eluate.

PCR products from pre-MDA samples were analysed by Bioanalyzer DNA 1000 Kit (Agilent, USA) and extracted DNA samples submitted to Source Bioscience (Cambridge, UK) for clean-up and sequencing. Post-MDA PCR products were analysed using 2% size select gel on the E-gel system (Thermo Fisher, USA). Desired bands were extracted and underwent clean-up with MinElute Reaction Cleanup Kit (Qiagen). DNA concentrations were assessed using HS DNA Kit on the Qubit 3.0 (Thermo Fisher) and adjusted to meet the Source Bioscience requirements. Sequencing analysis involved alignment to MG G37 strain to check for resistance-associated SNPs using Clustal Omega software.^17 18^

### Strain typing

As there was insufficient DNA for whole genome sequencing (WGS), we performed a validated dual locus sequence typing (DLST) method.^19^ Approximately 600 µL of residual swab eluate from MG-positive samples underwent DNA extraction using FastDNA SPIN Kit and the FastPrep 5G Instrument (MP Biomedicals, USA) according to manufacturer’s instructions, with the following modification: 600 µL eluate buffer was added to 600 µL 1% sodium dodecyl sulfate, 60 mM EDTA and 100 mM Tris buffer (pH8). DLST was performed using MG191 (*mgpB*) SNP typing combined with analysis of the MG309 variable number tandem repeat, as described previously.^3 19 20^ PCR was performed using Multiplex PCR Kit according to manufacturer’s instructions. Five microlitres of extracted DNA was used in each PCR reaction. If no amplicons were produced, DNA volume was increased to 15 µL. PCR was carried out on a T-100 thermocycler (Bio-Rad, USA): 95°C 15 min, 45 cycles of 94°C 30 s, 58°C 90 s, and 72°C 90 s, and a final extension of 72°C 10 min. PCR products underwent processing as described above for MG 23S rRNA genotyping of post-MDA samples. Where sequences were available for both loci for a sample, sequences were concatenated and underwent alignment by Clustal Omega.^17 18^ Phylogenetic trees were produced using RaxML^21^ and figures with FigTree V.1.4.3.^22^

### Sample size and statistical analysis

Sample size was constrained by the genital CT study design,^9^ which aimed to recruit a total of 375 women on the assumption of a change in prevalence from 20% to less than 10%. We assumed similar rates of infection and impact for MG. We did not anticipate identifying any azithromycin resistance pre-MDA due to azithromycin only being recommended for MDA for trachoma elimination in the Solomon Islands. Based on an estimated MG prevalence of 10% post-MDA and 12% azithromycin resistance development^14^ between pre-MDA and post-MDA, we expected to identify an additional two cases of azithromycin resistance post-MDA compared with pre-MDA. Logistic regression was used to calculate the ORs for factors associated with MG infection. Variables tested included patient demographics, symptoms, patient-reported previous treatment of other STIs and patient-reported receipt of MDA. Patients were not required to have a full data set to be included in the analysis and data were only excluded where they were missing for calculation of ORs (table 1). Analysis was carried out using Stata V.10.1 (StataCorp, USA). All sequences in this study have been submitted to EMBL-ENA (accession: PRJEB26624).

**Table 1:** Risk factors for *Mycoplasma genitalium* infection, including demographic data

Logistic regression risk factor analysis for being MG positive pre-MDA and post-MDA										
Pre-MDA						Post-MDA				
Univariate analysis						Univariate analysis				
Characteristic	Participants, n	With MG*, n (%)	OR	95% CI	P value	Participants, n	With MG*, n (%)	OR	95% CI	P value
Age group (years)										
15–24	68	9 (13.2)	1.00			113	13 (11.5)	1.00		
25–34	104	10 (9.6)	0.70	0.27 to 1.82	0.461	114	11 (9.7)	0.82	0.35 to 1.92	0.650
35–44	39	6 (15.4)	1.19	0.39 to 3.64	0.758	28	4 (14.3)	1.28	0.38 to 4.28	0.686
45-64†	11	0 (0)	–	–	–	0	0 (0)	–	–	–
Data unavailable	14					1				
Clinic										
K	94	11 (11.7)	1.00			77	10 (13.0)	1.00		
M	64	7 (10.9)	0.93	0.34 to 2.53	0.882	86	12 (14.0)	1.09	0.44 to 2.68	0.857
R	78	9 (12.0)	1.03	0.40 to 2.63	0.953	92	6 (6.5)	0.47	0.16 to 1.35	0.160
Data unavailable	0					1				
Ethnicity										
Melanesian	212	25 (11.8)	1.00			228	26 (11.4)	1.00		
Other	14	2 (14.3)	1.25	0.26 to 5.90	0.781	24	2 (8.3)	0.71	0.16 to 3.18	0.650
Data unavailable	10					4				
Urban/Rural										
Urban	214	22 (10.3)	1.00			197	23 (11.7)	1.00		
Rural	6	1 (16.7)	1.75	0.19 to 15.63	0.618	46	3 (6.5)	0.53	0.15 to 1.84	0.316
Data unavailable	16					13				
Education (years)										
	224	25 (10.7)	1.09	0.99 to 1.19	0.082	255	28 (11.0)	0.94	0.86 to 1.03	0.196
Data unavailable	12					1				
Currently married										
No	37	7 (18.9)	1.00			51	6 (11.8)	1.00		
Yes	192	19 (9.9)	0.47	0.18 to 1.22	0.120	198	21 (10.6)	0.89	0.34 to 2.33	0.813
Data unavailable	7					7				
Living with partner										
No	40	8 (20.0)	1.00			29	4 (13.8)	1.00		
Yes	184	17 (9.2)	0.41	0.16 to 1.02	0.056	217	23 (10.6)	0.74	0.24 to 2.32	0.606
Data unavailable	12					10				
STI in last 12 months										
No	224	25 (11.2)	1.00			229	23 (10.0)	1.00		
Yes	8	2 (25.0)	2.65	0.51 to 13.9	0.247	22	4 (18.2)	1.99	0.62 to 6.39	0.247
Data unavailable	4					5				
Symptoms										
No	188	23 (12.2)	1.00			222	26 (11.7)	1.00		
Yes	37	3 (8.1)	0.63	0.18 to 2.23	0.476	22	1 (4.5)	0.36	0.05 to 2.78	0.327
Data unavailable	11					12				
Gonorrhoea positive‡										
No	228	25 (11.0)	1.00			240	28 (11.7)	1.00		
Yes	5	2 (40.0)	5.41	0.86 to 33.98	0.072	15	0 (0)	–	–	–
Data unavailable	3					1				
TV positive§										
Negative	146	14 (9.6)	1.00			135	16 (11.9)	1.00		
Positive	87	13 (14.9)	1.66	0.74 to 3.71	0.220	120	12 (10.0)	0.83	0.37 to 1.83	0.637
Data unavailable	3					1				
Received MDA										
No	N/A	N/A	N/A	N/A	N/A	125	22 (17.6)	1.00		
Yes	N/A	N/A	N/A	N/A	N/A	126	5 (4.0)	0.19	0.07 to 0.53	0.001
Data unavailable						5				

Risk factor analysis for *M. genitalium* (MG) prevalence.

*MG positive defined by FTD Urethritis Plus Kit.

†Age group 45–64 years predicted failure perfectly.

‡Positive by BD ProbeTec and/or FTD Urethritis Plus Kit.

§Positive by FTD Urethritis Plus Kit.

MDA, mass drug administration; N/A, not applicable; TV, *Trichomonas vaginalis*.

## Results

### Population characteristics

**Figure 1:**
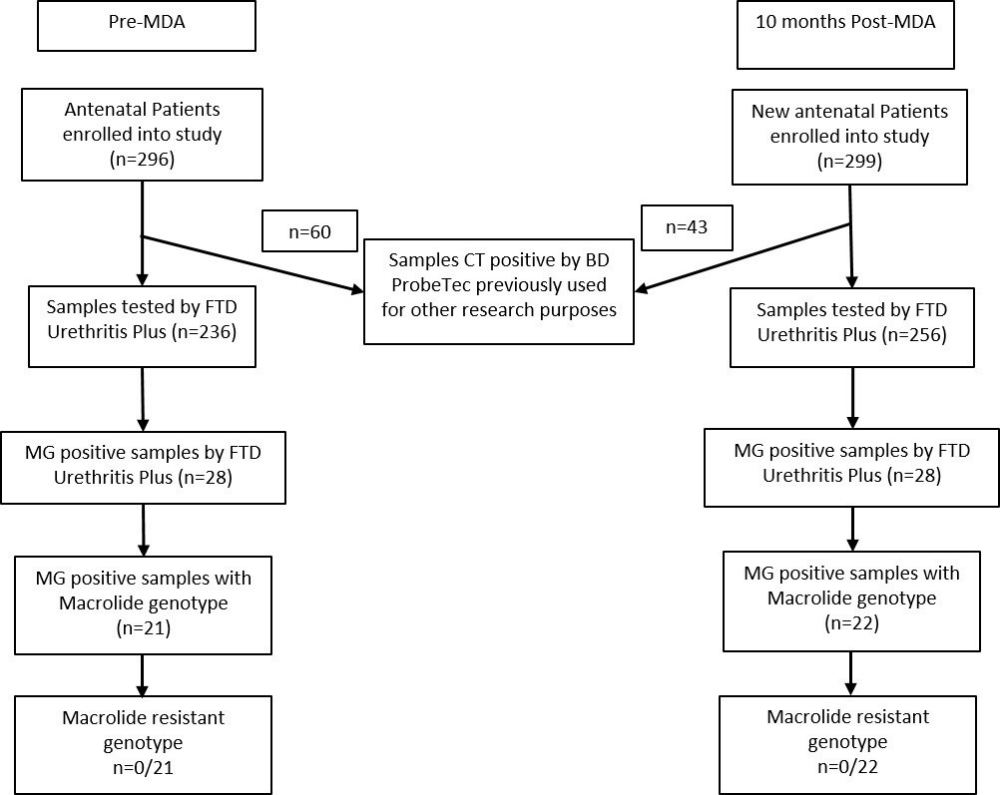
Sample flow. BD, Becton Dickinson; CT, *Chlamydia trachomatis*; FTD, Fast Track Diagnostics; MDA, mass drug administration; MG, *Mycoplasma genitalium*.

Figure 1 represents the pre-MDA and post-MDA sample flow. Table 1 displays the study population characteristics. Among pre-MDA and post-MDA patients included in the analysis, 38/236 (16.1%) vs 22/256 (8.6%; p=0.011) respectively reported having a symptom indicative of an STI (dyspareunia, abnormal vaginal discharge or genital ulcer) within the previous month. One hundred and twenty-six (49.2%) post-MDA patients reported receiving azithromycin as part of MDA; for four patients data were unavailable.

### Impact of MDA on MG prevalence and azithromycin resistance

No difference was found between pre-MDA and post-MDA MG prevalence; 11.9% (95% CI 8.3% to 16.6%; n=28/236) vs 10.9% (95% CI 7.9% to 15.4%; n=28/256) respectively. Azithromycin resistance genotypes were generated for 21/28 women pre-MDA and 22/28 women post-MDA (figure 1), no azithromycin resistance-conferring SNPs at the 2058 or 2059 positions of 23S rRNA were found at either time point.

### Risk factors for MG infection

No risk factors were significantly associated with MG infection pre-MDA. 17.6% (22/125) of women who reported not receiving MDA were found to be MG positive compared with 4% (5/126) of women who reported receiving MDA (OR of being MG positive after MDA receipt: 0.19 [p=0.001]). Despite post-MDA women having fewer STI symptoms than pre-MDA women, and being more likely to have been treated for an STI in the previous 12 months, these factors were not associated with reduced likelihood of being MG positive post-MDA.

### Strain typing

**Figure 2:**
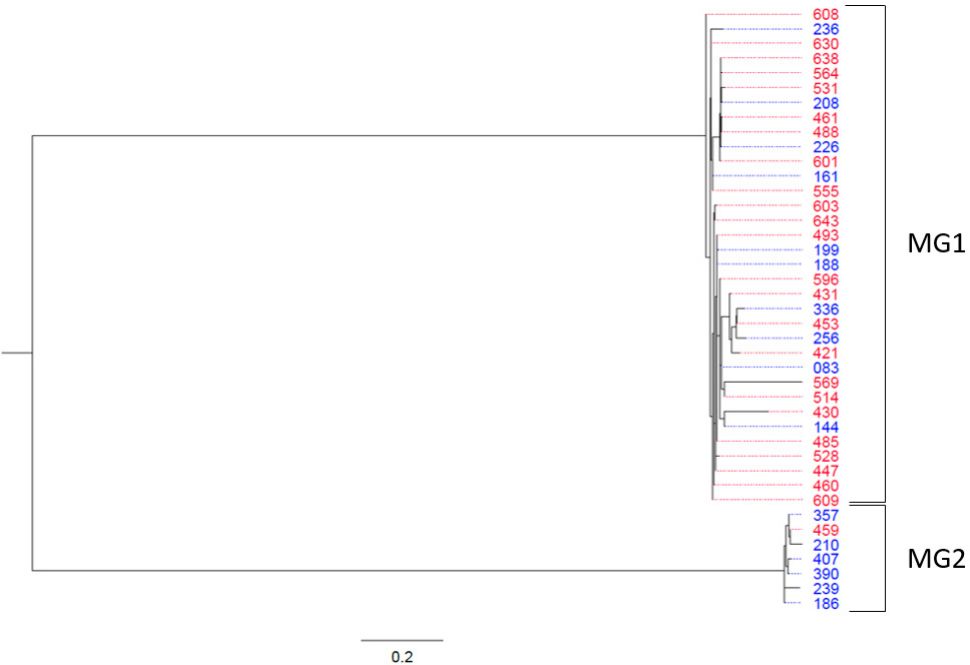
Phylogenetic tree of *M* *ycoplasma* *genitalium*-positive samples using dual locus sequence typing. Red denotes samples collected pre-MDA and blue denotes samples collected post-MDA; bootstrap value between MG1 and MG2=100%. Scale: mean number of nucleotide substitutions per site. MDA; mass drug administration.

Sequencing was successful for both MG191 and MG309 loci for 25/28 pre-MDA and 16/28 post-MDA samples. Sequence types diverged into two main lineages: MG1 (n=34) and MG2 (n=7) (bootstrap value 100%; figure 2). The proportion of MG2 lineage samples changed from 1/25 (4%) pre-MDA to 6/16 (37.5%) post-MDA (p<0.01; Fisher’s exact test), suggesting a degree of strain replacement between the time points. Overall, only 5/27 (18.5%) of post-MDA MG-positive patients received azithromycin, including only 2/10 MG1 and 0/5 MG2 DLSTs of those sequenced. Azithromycin treatment status was not available for one woman with MG2 strain type post-MDA. No other bootstrap values between individual MG infections were sufficiently high to confidently separate strains to any higher resolution.

## Discussion

In this secondary analysis of ANC attendees in the Solomon Islands, we found no change in prevalence of MG infection nor appearance of 23S rRNA genotypic markers of azithromycin resistance in the interval from before to 10 months after a single round of azithromycin MDA for trachoma elimination. We also demonstrated azithromycin receipt during MDA was associated with reduced likelihood of post-MDA MG positivity. A change in composition of MG strain types in those sampled was observed between pre-MDA and post-MDA patients.

Factors that may affect impact of MDA on STI transmission include antibiotic efficacy and treatment failure, MDA coverage especially in high STI-risk and STI-burdened populations and infection persistence. Contributions of these factors to reducing STI prevalence through direct treatment, or longer term by decreasing onward transmission, need to be considered in relation to the time period of MDA impact assessment.

Importantly, azithromycin receipt as part of the MDA was reported in only 49.2% of women post-MDA. Programme MDA coverage estimates are often unreliable as coverage is mostly calculated by doses given divided by estimated resident population, determined at the most recent census^23^; the denominator may be out of date or otherwise unrepresentative of the population present at the time of MDA. Furthermore, MDA coverage data are generally not available by age and sex. It was therefore not possible to compare MDA receipt between the study and wider populations. Neither data regarding sexual history between MDA receipt and study recruitment nor MDA receipt among sexual partners were collected. These data could have provided insight into potential reinfection risk and an explanation for why MG prevalence was unchanged between pre-MDA and post-MDA populations. The study was performed as two independent cross-sectional surveys within three ANCs, patients of which are perhaps at a different STI risk level than the general population. Importantly, sample size was determined by the genital CT study design, and therefore may not have been sufficient for detecting smaller overall prevalence changes or azithromycin resistance emergence. If the five MG-positive women who had reported MDA receipt post-MDA were positive because of persistence of infection following failure of azithromycin to cure infection, one might expect some of these to have developed azithromycin resistance. As no resistance-associated mutations were detected this would suggest that these patients were most likely infected post-MDA, consistent with high rates of transmission in the population and may also explain the high prevalence of infection. It was not possible to sequence a proportion of the MG-positive samples (7/28 [25%] pre-MDA; 6/28 [21.4%] post-MDA) for 23S mutations, and it is possible that these samples contained macrolide-resistant strains. It cannot be discounted that those we were unable to sequence were not true positives. Unfortunately, there are no published sensitivity and specificity data on FTDUP performance for detection of MG sensitivity and specificity. In another body of work we found FTDUP sensitivity to be 100% and specificity to be 95.8%, albeit in a limited sample size of 122 (unpublished data). Therefore, we do not consider test performance to potentially be a major limiting factor in correctly identifying MG-positive and negative samples. Finally, it was not possible to test CT positives for MG, due to their allocation to another study, which may have led to an underestimate of MG positivity both pre-MDA and post-MDA given that coinfection with CT and MG is common.^24–26^ Additionally, as a significant reduction in CT prevalence was found in the primary analysis, exclusion of CT positives (n=103, pre-MDA n=60, post-MDA n=43) may be a source of bias as a greater proportion of CT positives was excluded from the pre-MDA samples versus the post-MDA samples.

Despite these limitations, our risk factor analysis provides strong evidence that those receiving azithromycin MDA had reduced odds of being MG positive, implying that increasing MDA coverage may be key in achieving greater MG prevalence impact, at least in the short term. Significant scope for increasing MDA uptake in this population exists as only 49.2% of women recruited post-MDA reported azithromycin receipt. Post-MDA enrolment occurred approximately 10 months after baseline, making it unlikely that women enrolled at this time point would have been pregnant during MDA, an explanation that might otherwise account for low azithromycin uptake. However, we cannot discount the possibility that some women avoided MDA through fear of taking treatment while trying to conceive. It is unclear whether 10 months post-MDA is the most appropriate time point to measure MDA impact on prevalence, as any initial MG prevalence reduction may have waned by 10 months. In remote island populations, this might be expected to occur if there were relatively high rates of sexual transmission of endogenous, rather than imported, MG infection. More data on transmission dynamics and STI epidemiology within the Solomon Islands would help to establish the most appropriate time points for measuring MDA impact on STI prevalence.

These data may also help identify factors associated with MG infection. Our risk factor analysis did not identify any factors other than self-reported MDA receipt, despite high prevalences pre-MDA (11.9%) and post-MDA (10.9%). This may be due to the relatively small sample size, or the variables collected not including factors that are important MG infection correlates in this population. Risk factor analyses are useful in helping target prevention and control strategies and interventions, but there are limited data on MG prevalence in pregnant women. The high prevalence of CT and *N. gonorrhoeae* previously reported^9^ and MG in our study, both pre-MDA and post-MDA, indicates that this population would benefit from effective STI prevention strategies. Further epidemiological investigations in different population groups are warranted to help develop, monitor and evaluate these strategies, including assessing associations between MG infection and adverse pregnancy outcomes.

It is possible that single-dose azithromycin used during MDA was not as effective against MG as against CT, explaining the lack of overall impact on MG prevalence. MG cure rates with 1 g azithromycin are known to be as low as 81%, independent of pre-existing macrolide resistance,^27^ much lower than CT cure rates.^28^ However, as strong evidence existed of reduced likelihood of being MG positive in those who reported MDA receipt, we do not believe this to be a major explanation for our results.

We were unable to perform WGS for strain typing because of insufficient DNA. However, WGS for MG has not yet been validated for phylogenetic analysis, with concerns raised about degrees of genome recombination.^29^ Using DLST, we identified two major strain lineages, as well as detected an increase in MG2 proportion post-MDA. This shift might have occurred due to differences in characteristics of infection such as bacterial load, leading to variable antibiotic susceptibility,^30^ which may introduce sequencing bias. No post-MDA patients with MG2 and only 2/10 with MG1 reported receiving azithromycin, suggesting low likelihood of strain replacement occurring directly because of azithromycin receipt. However, it is possible that MDA effects on strain representation in the general population may have been transmitted to the ANC population. Whether this is measurable 10 months after MDA is questionable and other factors, such as natural bacterial evolution, changes in sexual networks over time, or stochastic changes in a relatively small effective population size, may account for these findings.

Absence of azithromycin resistance in MG in the Solomon Islands contrasts with high resistance prevalence in other parts of the Western Pacific Region, namely Australia and Japan.^31 32^ The Solomon Islands has low azithromycin usage, with MDA for trachoma elimination being the only recommended use within the country at the time of MDA. In contrast, in other, especially high-income, countries, azithromycin is indicated for a number of conditions, including respiratory infections.^33^ MG has only one 23S rRNA locus making it particularly susceptible to selection of resistant strains, which occurs in approximately 12% of treated patients.^14^ Cure rates are observed to decline where 1 g is part of the national standard for STI treatment.^14 30^ We may therefore have expected to observe an increased prevalence of azithromycin resistance markers being selected given MDA coverage. However, relatively low coverage in the ANC population combined with relatively low sample size may explain why we did not detect emergent AMR. It is possible that repeat MDA rounds may select for resistance through increased selection pressure, undermining the possible benefits of actual receipt of azithromycin during MDA that we observed in this round. More studies focusing on general populations and over multiple MDA rounds would more fully evaluate the risk of STI AMR development following MDA.

In this first study, assessing the impact of a single round of azithromycin MDA for trachoma elimination on MG prevalence and AMR among ANC attendees in an island population, we did not detect reduction in MG prevalence nor the appearance of azithromycin resistance following a single round of MDA with 1 g azithromycin for trachoma elimination. However, receipt of azithromycin was associated with reduced odds of being MG positive post-MDA. A number of factors may account for these findings, including low MDA coverage, high reinfection risk from untreated partners, insufficient sample size, time gap between pre-MDA and post-MDA sample collection and perhaps a lack of 1 g azithromycin efficacy for treatment of MG. These findings cannot otherwise be confidently explained by an observed change in strain representation between pre-MDA and post-MDA sample sets. Benefits of MDA for trachoma elimination, and in national programmes for other neglected tropical diseases, must continue to be weighed against potential negative consequences, such as AMR emergence. Further investigation of the impact of multiple rounds of azithromycin MDA on MG prevalence and AMR in different populations and settings is warranted.

**Key messages**

- First study to investigate the impact of mass drug administration (MDA) for trachoma elimination on *Mycoplasma genitalium* prevalence and azithromycin resistance.
- In this Solomon Islands’ antenatal care population, MDA with self-reported coverage of approximately 50% did not appear to impact *M. genitalium* prevalence or azithromycin resistance.
- However, in those who reported receiving azithromycin as part of MDA, we found reduced odds of *M. genitalium* infection.
- Overall, there was evidence suggestive of *M. genitalium* strain replacement following MDA.

## References

[1] Haggerty CL, Taylor BD Mycoplasma genitalium : An Emerging Cause of Pelvic Inflammatory Disease. Infect Dis Obstet Gynecol 2011;2011:1–9. 10.1155/2011/959816 PMC3253449 22235165

[2] Jensen JS Mycoplasma genitalium: the aetiological agent of urethritis and other sexually transmitted diseases. J Eur Acad Dermatol Venereol 2004;18:1–11. 10.1111/j.1468-3083.2004.00923.x 14678525

[3] Pond MJ, Nori AV, Witney AA, et al High prevalence of antibiotic-resistant Mycoplasma genitalium in nongonococcal urethritis: the need for routine testing and the inadequacy of current treatment options. Clin Infect Dis 2014;58:631–7. 10.1093/cid/cit752 24280088 PMC3922211

[4] Jensen JS, Cusini M, Gomberg M, et al 2016 European guideline on Mycoplasma genitalium infections. J Eur Acad Dermatol Venereol 2016;30:1650–6. 10.1111/jdv.13849 27505296

[5] Bourne RRA, Stevens GA, White RA, et al Causes of vision loss worldwide, 1990-2010: a systematic analysis. Lancet Glob Health 2013;1:e339–49. 10.1016/S2214-109X(13)70113-X 25104599

[6] Solomon AW, Zondervan M, Kuper H, et al Trachoma control: a guide for programme managers. Geneva, Switzerland: World Health Organisation, 2006.

[7] O'Brien KS, Emerson P, Hooper PJ, et al Antimicrobial resistance following mass azithromycin distribution for Trachoma: a systematic review. Lancet Infect Dis 2019;19:e14–25. 10.1016/S1473-3099(18)30444-4 30292480

[8] Evans JR, Solomon AW, Cochrane Eyes and Vision Group Antibiotics for trachoma. Cochrane Database Syst Rev 2011;354 10.1002/14651858.CD001860.pub3 21412875

[9] Marks M, Bottomley C, Tome H, et al Mass drug administration of azithromycin for trachoma reduces the prevalence of genital Chlamydia trachomatis infection in the Solomon Islands. Sex Transm Infect 2016;92:261–5. 10.1136/sextrans-2015-052439 26888658 PMC4893086

[10] Keenan JD, Bailey RL, West SK, et al Azithromycin to reduce childhood mortality in sub-Saharan Africa. N Engl J Med 2018;378:1583–92. 10.1056/NEJMoa1715474 29694816 PMC5849140

[11] Trembizki E, Buckley C, Bletchly C, et al High levels of macrolide-resistant Mycoplasma genitalium in Queensland, Australia. J Med Microbiol 2017;66:1451–3. 10.1099/jmm.0.000584 28893363 PMC5845567

[12] Couldwell DL, Jalocon D, Power M, et al Mycoplasma genitalium: high prevalence of resistance to macrolides and frequent anorectal infection in men who have sex with men in western Sydney. Sex Transm Infect 2018;94:406–10. 10.1136/sextrans-2017-053480 29567802

[13] Deguchi T, Ito S, Yasuda M, et al Surveillance of the prevalence of macrolide and/or fluoroquinolone resistance-associated mutations in Mycoplasma genitalium in Japan. J Infect Chemother 2018;24:861–7. 10.1016/j.jiac.2018.08.009 30190106

[14] Horner P, Ingle SM, Garrett F, et al Which azithromycin regimen should be used for treating Mycoplasma genitalium? A meta-analysis. Sex Transm Infect 2018;94:14–20. 10.1136/sextrans-2016-053060 28717050

[15] Jensen JS, Bradshaw CS, Tabrizi SN, et al Azithromycin treatment failure in Mycoplasma genitalium-positive patients with nongonococcal urethritis is associated with induced macrolide resistance. Clin Infect Dis 2008;47:1546–53. 10.1086/593188 18990060

[16] International trachoma initiative, 2018 Available: www.trachoma.org

[17] Sievers F, Wilm A, Dineen D, et al Fast, scalable generation of high-quality protein multiple sequence alignments using Clustal omega. Mol Syst Biol 2011;7 10.1038/msb.2011.75 PMC3261699 21988835

[18] Goujon M, McWilliam H, Li W, et al A new bioinformatics analysis tools framework at EMBL-EBI. Nucleic Acids Res 2010;38:W695–W699. 10.1093/nar/gkq313 20439314 PMC2896090

[19] Cazanave C, Charron A, Renaudin H, et al Method comparison for molecular typing of French and Tunisian Mycoplasma genitalium-positive specimens. J Med Microbiol 2012;61:500–6. 10.1099/jmm.0.037721-0 22160316

[20] Hjorth SV, Björnelius E, Lidbrink P, et al Sequence-based typing of Mycoplasma genitalium reveals sexual transmission. J Clin Microbiol 2006;44:2078–83. 10.1128/JCM.00003-06 16757601 PMC1489459

[21] Stamatakis A RAxML-VI-HPC: maximum likelihood-based phylogenetic analyses with thousands of taxa and mixed models. Bioinformatics 2006;22:2688–90. 10.1093/bioinformatics/btl446 16928733

[22] Rambaut A Figtree 1.4.3, 2016 Available: http://tree.bio.ed.ac.uk/software/figtree/

[23] Cromwell EA, Ngondi J, McFarland D, et al Methods for estimating population coverage of mass distribution programmes: a review of practices in relation to trachoma control. Trans R Soc Trop Med Hyg 2012;106:588–95. 10.1016/j.trstmh.2012.07.011 22884927

[24] Huppert JS, Mortensen JE, Reed JL, et al Mycoplasma genitalium detected by transcription-mediated amplification is associated with Chlamydia trachomatis in adolescent women. Sex Transm Dis 2008;35:250–4. 10.1097/OLQ.0b013e31815abac6 18490867 PMC3807598

[25] Tosh AK, Van Der Pol B, Fortenberry JD, et al Mycoplasma genitalium among adolescent women and their partners. J Adolesc Health 2007;40:412–7. 10.1016/j.jadohealth.2006.12.005 17448398 PMC1899169

[26] Broad CE, Harding-Esch E, Harrison M, et al O12.2 co-infection and macrolide antimicrobial Resistance (AMR) of Mycoplasma genitalium with Neisseria gonorrhoeae and Chlamydia trachomatis, in females, heterosexual males, and men-who-have-sex-with-men. Sex Transm Infect 2017;93(Suppl 2):A27.1–A27.

[27] Jensen JS, Bradshaw C Management of Mycoplasma genitalium infections - can we hit a moving target? BMC Infect Dis 2015;15 10.1186/s12879-015-1041-6 PMC4545773 26286546

[28] Kong FYS, Tabrizi SN, Law M, et al Azithromycin versus Doxycycline for the treatment of genital Chlamydia infection: a meta-analysis of randomized controlled trials. Clin Infect Dis 2014;59:193–205. 10.1093/cid/ciu220 24729507

[29] Fookes MC, Hadfield J, Harris S, et al Mycoplasma genitalium: Whole genome sequence analysis, recombination and population structure. BMC Genomics 2017;18 10.1186/s12864-017-4399-6 PMC5745988 29281972

[30] Walker J, Fairley CK, Bradshaw CS, et al Mycoplasma genitalium incidence, organism load, and treatment failure in a cohort of young Australian women. Clin Infect Dis 2013;56:1094–100. 10.1093/cid/cis1210 23300236

[31] Murray GL, Bradshaw CS, Bissessor M, et al Increasing Macrolide and Fluoroquinolone Resistance in Mycoplasma genitalium. Emerg Infect Dis 2017;23:809–12. 10.3201/eid2305.161745 28418319 PMC5403035

[32] Kikuchi M, Ito S, Yasuda M, et al Remarkable increase in fluoroquinolone-resistant Mycoplasma genitalium in Japan. J Antimicrob Chemother 2014;69:2376–82. 10.1093/jac/dku164 24894419

[33] British National Formulary Azithromycin. Available: https://bnf.nice.org.uk/drug/azithromycin.html

